# Deep-Precognitive Diagnosis: Preventing Future Pandemics by Novel Disease Detection With Biologically-Inspired Conv-Fuzzy Network

**DOI:** 10.1109/ACCESS.2022.3153059

**Published:** 2022-02-21

**Authors:** Aviral Chharia, Rahul Upadhyay, Vinay Kumar, Chao Cheng, Jing Zhang, Tianyang Wang, Min Xu

**Affiliations:** Mechanical Engineering DepartmentThapar Institute of Engineering and Technology 29080 Patiala Punjab 147004 India; Electronics and Communication Engineering DepartmentThapar Institute of Engineering and Technology 29080 Patiala Punjab 147004 India; Department of MedicineBaylor College of Medicine 3989 Houston TX 77030 USA; Department of Computer ScienceUniversity of California at Irvine 8788 Irvine CA 92697 USA; Department of Computer Science and Information TechnologyAustin Peay State University 2536 Clarksville TN 37044 USA; Computational Biology DepartmentSchool of Computer ScienceCarnegie Mellon University 6612 Pittsburgh PA 15213 USA; Computer Vision DepartmentMohamed bin Zayed University of Artificial Intelligence Abu Dhabi United Arab Emirates

**Keywords:** Deep learning, COVID-19, medical imaging, computer-aided diagnosis, pandemics

## Abstract

Deep learning-based Computer-Aided Diagnosis has gained immense attention in recent years due to its capability to enhance diagnostic performance and elucidate complex clinical tasks. However, conventional supervised deep learning models are incapable of recognizing novel diseases that do not exist in the training dataset. Automated early-stage detection of novel infectious diseases can be vital in controlling their rapid spread. Moreover, the development of a conventional CAD model is only possible after disease outbreaks and datasets become available for training (viz. COVID-19 outbreak). Since novel diseases are unknown and cannot be included in training data, it is challenging to recognize them through existing supervised deep learning models. Even after data becomes available, recognizing new classes with conventional models requires a complete extensive re-training. The present study is the *first* to report this problem and propose a novel solution to it. In this study, we propose a new class of CAD models, i.e., Deep-Precognitive Diagnosis, wherein artificial agents are enabled to identify unknown diseases that have the potential to cause a pandemic in the future. A *de novo* biologically-inspired Conv-Fuzzy network is developed. Experimental results show that the model trained to classify Chest X-Ray (CXR) scans into normal and bacterial pneumonia detected a novel disease during testing, unseen by it in the training sample and confirmed to be COVID-19 later. The model is also tested on SARS-CoV-1 and MERS-CoV samples as unseen diseases and achieved state-of-the-art accuracy. The proposed model eliminates the need for model re-training by creating a new class in real-time for the detected novel disease, thus classifying it on all subsequent occurrences. *Second,* the model addresses the challenge of limited labeled data availability, which renders most supervised learning techniques ineffective and establishes that modified fuzzy classifiers can achieve high accuracy on image classification tasks.

## I. Introduction

Throughout human history, there have been several pandemics, the most recent and ongoing being the SARS-CoV-2. The first case of the disease was reported in late December 2019 in Wuhan, China [1], [2].

Ever since the first case of COVID-19 was reported, the world has seen over 219 million cases and 4.547 million deaths due to the virus [3]. Despite tremendous improvements in our understanding of microbial hazards over the last two decades, humans remain as vulnerable as ever to unexpected attacks by unknown viruses. The World Health Organization (WHO) has adopted ‘Disease X’ as a placeholder name for hypothetical, unknown diseases that might trigger a future epidemic. This list is periodically updated and includes diseases such as Middle East-respiratory syndrome coronavirus (MERS-CoV) and Severe Acute Respiratory Syndrome (SARS), among others. Past studies [4] suggest that the increasing scale of anthropogenic destruction of biodiversity is paving the way to a ‘pandemic era’. In such a situation, it is projected that roughly 850,000 distinct viruses might spread from animals to people, with SARS-CoV-2 being just one of them [4]. The study has been strengthened by the increased frequency with which novel disease outbreaks have occurred in the last two decades. This has prompted an urgent need for reconsideration, as well as a need for substantial actions to be implemented to address a health catastrophe of such magnitude. Table 1 enlists various recent novel zoonotic virus outbreaks [5]–[7].

**TABLE 1:** Increased Rate of Spread of Numerous Novel Zoonotic Viruses in Recent Disease Outbreaks, Suggestive of the Approaching $`Pandemic~era'$. Data Taken From [5]–[7]

Disease	First Reported	Rate of Spread	Time period	Total Infected	Total Deaths	Fatality (%)	Countries Affected
SARS-CoV-1	Feb 2003, Foshan, China	High	Major outbreak lasted eight months; declared contained in July 2003; but cases reported till date	8096	774	9.6%	29 Countries
MERS-CoV	June 2012, Jeddah, Saudi Arabia	Low	June 2012 - till date	2574	885	34.4%	27 Countries
SARS-CoV-2	Dec 2019, Wuhan, China	Very High	Dec, 2019 - till date	219 Million	4.547 Million	2–3%	Worldwide (Pandemic)

### A. Background

Presently, numerous researchers are involved in developing new methods for rapid disease detection to increase the rate of daily tests. Real-time Reverse-transcription polymerase chain reaction (rRT-PCR) [8] is the most widely used test for diagnosing COVID-19. However, many times, the test fails to detect the disease in case of a newly evolved coronavirus strain before extracting the new virus’s DNA sequence, potentially delaying testing [9]. Today, deep learning (DL) has been used in a wide range of areas which includes developing solutions for aiding the visually impaired [10], solving a 50-year-old grand protein folding challenge [11], analyzing macromolecules from cellular electron Cryo-tomography [12], [13], developing complex intrusion-based detection systems [14]–[18], enabling IoT-based systems [19], [20], etc. DL-based computer-aided diagnosis (CAD) has drawn immense attention in recent years for its capability to enhance diagnosis performance and elucidate complex clinical tasks.

**FIGURE 1.**
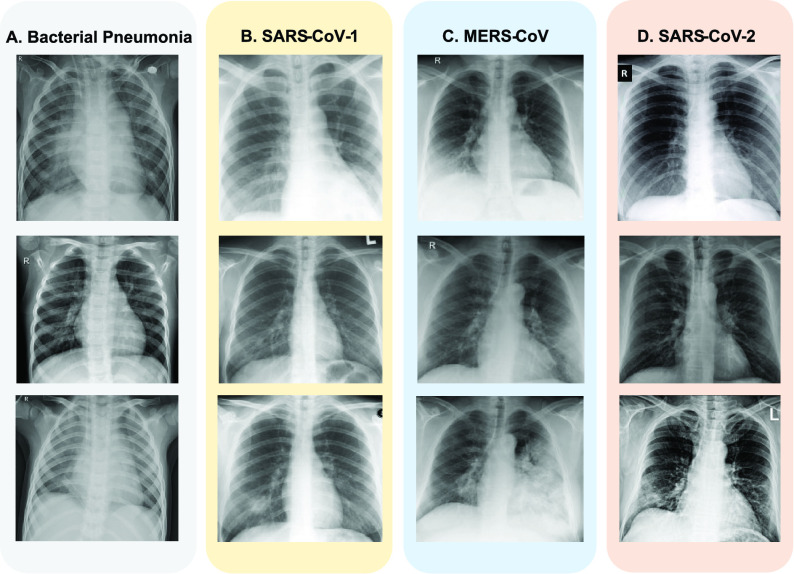
Representative images from the dataset [48], [49] showing Anteroposterior CXR scans of patients diagnosed with (a) Bacterial Pneumonia (b) SARS-CoV-1 (c) MERS-CoV (d) SARS-CoV-2 caused by the novel coronavirus (n-CoV).

During the pandemic, researchers have focused on developing various computational models for the rapid detection of SARS-CoV-2 on CXR scans with high accuracy and sensitivity [21]–[26]. Initial analysis of patients with severe symptoms showed signs of pneumonia [27]. Others have attempted to predict disease severity from X-rays. Figure 1 shows CXR scans of patients infected with various respiratory diseases- bacterial pneumonia, SARS-CoV-1, MERS-CoV, and SARS-CoV-2 (or COVID-19). The use of computed tomography (CT) is another way for detecting COVID-19 infection. However, the high radiation doses (also comparatively higher costs) makes it unsuitable for usage, particularly for pregnant women and children [28].

### B. Motivation

Although these methods help and provide the radiologist with assistance, they act only after a pandemic outbreak has occurred. Presently, little/ no study exists on developing a model that can detect novel diseases that have the potential to cause a future pandemic at their preliminary stage of spread itself. Such a model acts before a pandemic outbreak. This task, nevertheless, remains challenging.

Other questions that remain unaddressed and hinders the development of next-generation CAD models [29] include, *first*, all supervised learning models rely heavily on the availability of labeled medical datasets. Since highly skilled experts perform their collection, it requires considerable time and resources. Moreover, it is difficult to gather a large dataset of positive disease samples in an early stage of dissemination [29]. Therefore, an accurately labeled dataset can be available only after the disease outbreak has taken place, its multiple incidences have been reported, researched upon, and its pathological tests are available. A future disease outbreak may be a highly infectious one (viz. SARS-CoV-2) and spread at an even faster rate. Such an outbreak may not provide time to re-develop and deploy CAD models. *Second*, the task of detecting novel diseases is hard to visualize as a supervised learning problem in the absence of labeled training cases. *Third*, each time a novel disease outbreak ensues, new models need to be developed and updated throughout the medical system, following a similar pipeline. *Fourth*, many DL X-ray/ CT-scan models have low accuracy over cross-validation datasets [30], possibly since these were trained on a single dataset. *Fifth*, the emergence of newly mutated viruses results in a major change in the dataset, severely restricting the performance of conventional classification-based approaches. These complexities pose significant challenges in the development of a next-generation CAD model (one which aims to detect a novel disease before its outbreak) and motivates the research community towards this problem.

### C. Contributions

In the present study, we highlight the utility of the proposed model with the hypothetical outbreak of three pathogens (SARS-CoV-1, MERS-CoV, and COVID-19) that are unseen by the model, i.e., ‘Disease X’ as suggested by the WHO to be a potential cause of a future major epidemic. Along with COVID-19 as the unseen disease, the efficacy of the proposed model is demonstrated experimentally on SARS-CoV-1 and MERS-CoV, present in WHO’s list. Here it has been assumed that a future pandemic might be caused by a novel coronavirus on the similar lines of previous disease outbreaks, impacting lungs, and could be captured through CXR scans. The significant contributions of this paper include-

• The concept of ‘Deep-Precognitive diagnosis’ is introduced as a new class of CAD having broad applicability in developing future diagnosis models capable of detecting novel diseases at preliminary stage of spread.
• A novel solution is proposed for the introduced problem. The task is formulated as a class membership lookup problem using a biologically-inspired Conv-fuzzy network. The model’s ability to detect occurrences of novel diseases on CXR scans with state-of-the-art (SOTA) accuracy, is shown on three coronaviruses, as unseen diseases.
• The proposed algorithm eliminates the need for re-training for each new disease outbreak. Real-time architecture update enables new class creation for the detected novel disease, classifying it on all further occurrences.
• The challenge of limited labeled data availability is addressed. The proposed model has the ability to learn complex feature space on small datasets, unlike DL models. Further, as the architecture is semi-supervised, a labeled dataset is not needed for every sample, reducing heavy dependence on labeled datasets. Unlike conventional models, the proposed model requires single-pass training. Thus, there is a massive reduction in training time is observed.
• According to our knowledge, the present work is the *first* to adapt Fuzzy reflex classifiers for image classification tasks and quantitatively establish that they achieve comparable performance to SOTA ML classifiers.

The remaining paper is organized as follows. Section II introduces the relevant related works. Section III describes the proposed model, its architecture design, and algorithm. The experimental settings, experiments performed, and the results are elaborated in Section IV. This is followed by the discussion in Section V. Section VI discusses the limitations and the future work of the study. Finally, the conclusion is presented in Section VII.

## II. Related Work

### A. Manual Detection of Novel Diseases

Presently, computed tomography (CT) scans offering 3D anatomy are examined by expert radiologists to find abnormal features in the thoracic region suggestive of novel diseases, based on the type, and extent of lesions [31]. These are further sent for clinical tests to confirm new disease presence. Even for detection of COVID-19, firstly most common manifestation and patterns of lung abnormality were used for manual classification [32]. The initial screening is mainly dependent on expert radiologists that may not be present in all diagnostic centers. Presently, as per our knowledge, there exists no end-to-end model that has been proposed to address the problem. If compared with CT-scans, CXR scans do not provide three-dimensional anatomy, but pneumonia and most other diseases can be differentiated, though many Radiologists consider CXR scans as “the most challenging plain film to interpret correctly” [33]. However, due to its fast imaging speed, low radiation, and low cost, X-rays are the most widely used CAD imaging modality. Thus, X-rays have been used in this study to demonstrate the effectiveness of the proposed model.

### B. Deep Learning Breakthrough in CAD

Today, DL-based CAD has been extensively used to improve the accuracy of prediction for screening both infectious and non-infectious diseases [34]. The development of the first CAD model traces back to 1976 when the first CAD model called MYCIN was developed. It used 450 rules designed for bacterial infection and suggested antibiotics to patients [35]. The recent development of DL has triggered a new era in CAD, including breakthroughs in detection, delineation of pathological abnormalities, disease progression monitoring, and therapy response. Many such models have been developed by researchers for classification of various communicable diseases including SARS, EBOLA [36], HIV [37], [38], COVID-19 [23], [39] among others.

### C. Challenges in Next-Generation Diagnosis

Nearly all CAD models involve three sub-steps: data feature extraction (in medical image analysis), their classification, and the diagnosis. However, presently, many challenges are faced in CAD development that inhibits the progress in this field of research [29]. The *first* major challenge is the availability of adequately labeled medical datasets for novel disease outbreaks. While the quantity of Electronic health records (EHRs) has increased by multiple folds due to proper data collection; data records are frequently missing, recorded incorrectly, or improperly disposed of [40]. *Second*, it is difficult to get positive samples in an early stage of novel disease outbreak. Annotating such samples further requires great expertise. Studies by [41], [42] shows that general intuition of better classifier performance for large training dataset is misleading and models can attain good accuracy on limited datasets. But the development of CAD models that can work on limited dataset still remains a challenging task. *Third*, the emergence of newly mutated viruses results in a significant change in the dataset, severely restricting the performance of classification-based approaches.

### D. Fuzzy Logic in Computer-Aided Diagnosis

Even though the study and application of fuzzy logic has received immense attention in recent years [43]–[45], the use of fuzzy logic for classification in CAD models is an open research area [29]. Fuzzy min-max neural networks, first introduced by [46], have been used in many applications. Various improvements have been proposed [47] in the original network to improve the model’s overall accuracy. One of the areas of past work includes fuzzy reflex classifiers [48], where a self-supervised network learns on data and classifies it. However, this network has not been investigated for tasks involving high-dimensional data like image classification, including medical image classification in CAD models. We demonstrate how the recent rise of DL-based supervised feature extraction bridges the gap between fuzzy reflex classifiers and their machine learning (ML) counterparts.

### E. Deep Learning for Novel Disease Detection

Today, very few works focus on developing CAD models capable of detecting unseen novel diseases. This problem has the potential for future pandemic prevention and control. Recent attempts have been made by [49] on how to learn classifiers to predict or choose to defer the classification decision to a downstream expert. Work by [50] has investigated unseen disease detection using DL on CXR scans, but the study is limited to an internal validation set without an external test set including different unseen diseases. Moreover, the proposed model architecture cannot be updated in real-time and has to be re-developed for adding new classes each time a novel disease is detected.

## III. Proposed Methodology

### A. Problem Formulation

In traditional models, an input test sample $a_{h}$, is classified amongst the previously learnt classes $C_{i=1\ldots k}$. These classification models are based on a ‘closed world’ assumption [50], where no new classes are present in the test set, which hardly is the case for real-world medical applications. Therefore, such models suffer from a major disadvantage, i.e., samples belonging to a new class (i.e., high probability of a novel disease), which the model has not seen or not been trained on previously, get wrongly classified in one of the existing classes, leading to diagnostic failure. Moreover, for every introduction of a new disease, these models need to be re-trained and developed.

**FIGURE 2.**
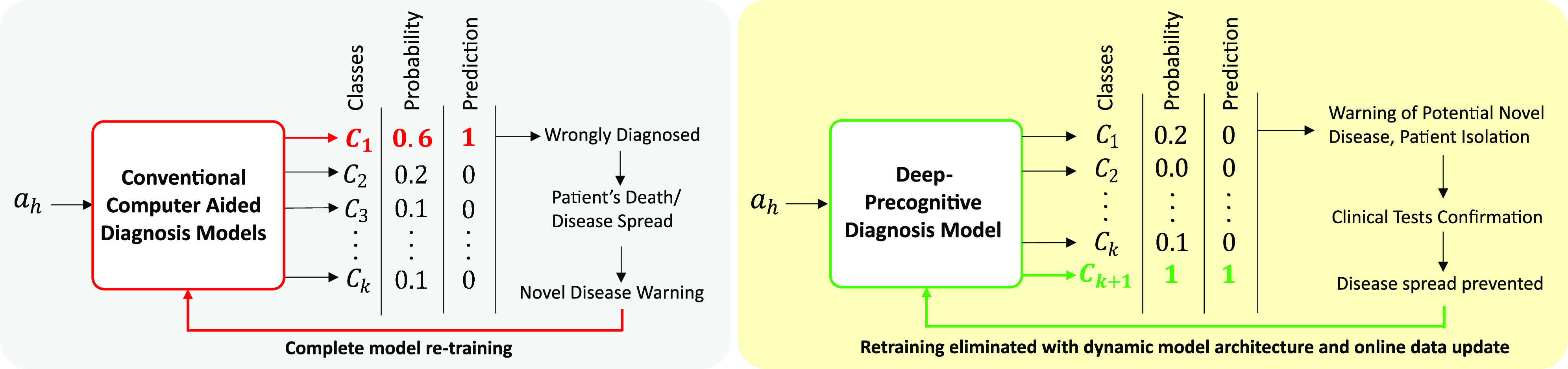
(a) Traditional CAD models are based on a “closed world” assumption, where no new classes are present in the test set. This is hardly the case for real-world medical applications. These models are limited to classify input test sample $a_{h}$ which may belong to a new class of disease as one amongst the previously learnt classes $C_{i=1\ldots k}$ having the highest probability. This leads to wrong diagnosis which may cause disease spread/ patient’s death. Even after clinical tests establish existence of a new disease, to classify it, complete model retraining is required (b) Deep-Precognitive diagnosis detects this novel disease, creates new class $C_{k+1}$ in real time and classifies the novel disease to this class. The model’s accuracy tend to increase with time over this new class as it sees its more samples. Therefore, the need for model re-training is eliminated.

Unlike conventional models, Deep-Precognitive diagnosis (DPD) refers to developing intelligent CAD models that can detect the occurrences of new diseases at their preliminary stage of spread. The proposed model (refer Figure 2(b)) has the ability to detect new disease classes and create a new class $C_{k+1}$ for them in real-time. Such a model will have the capability to control the spread of novel diseases with an early warning of a future pandemic. Figure 2 shows the conventional CAD models in contrast with the proposed Deep-Precognitive diagnosis model.

DPD is difficult to be thought of as a supervised learning problem. Since the dataset of the novel disease, which the model is trying to detect, cannot be available previously. Instead of a supervised learning problem, we frame the task as a class membership lookup problem to solve it. For each input, the model learns both contrasting and similar visual features between different classes $C_{i=1\ldots k}$. The input feature vector $a_{h}$ is mapped in a high dimensional feature space (here, $n= 512$ dimensional) where its classification becomes feasible. This is particularly different from Convolutional Neural Network (CNN) [51] based classification, which focuses mainly on learning those image features which can potentially be useful in distinguishing between two classes.

### B. Disease Feature Extraction and Mapping

**FIGURE 3.**
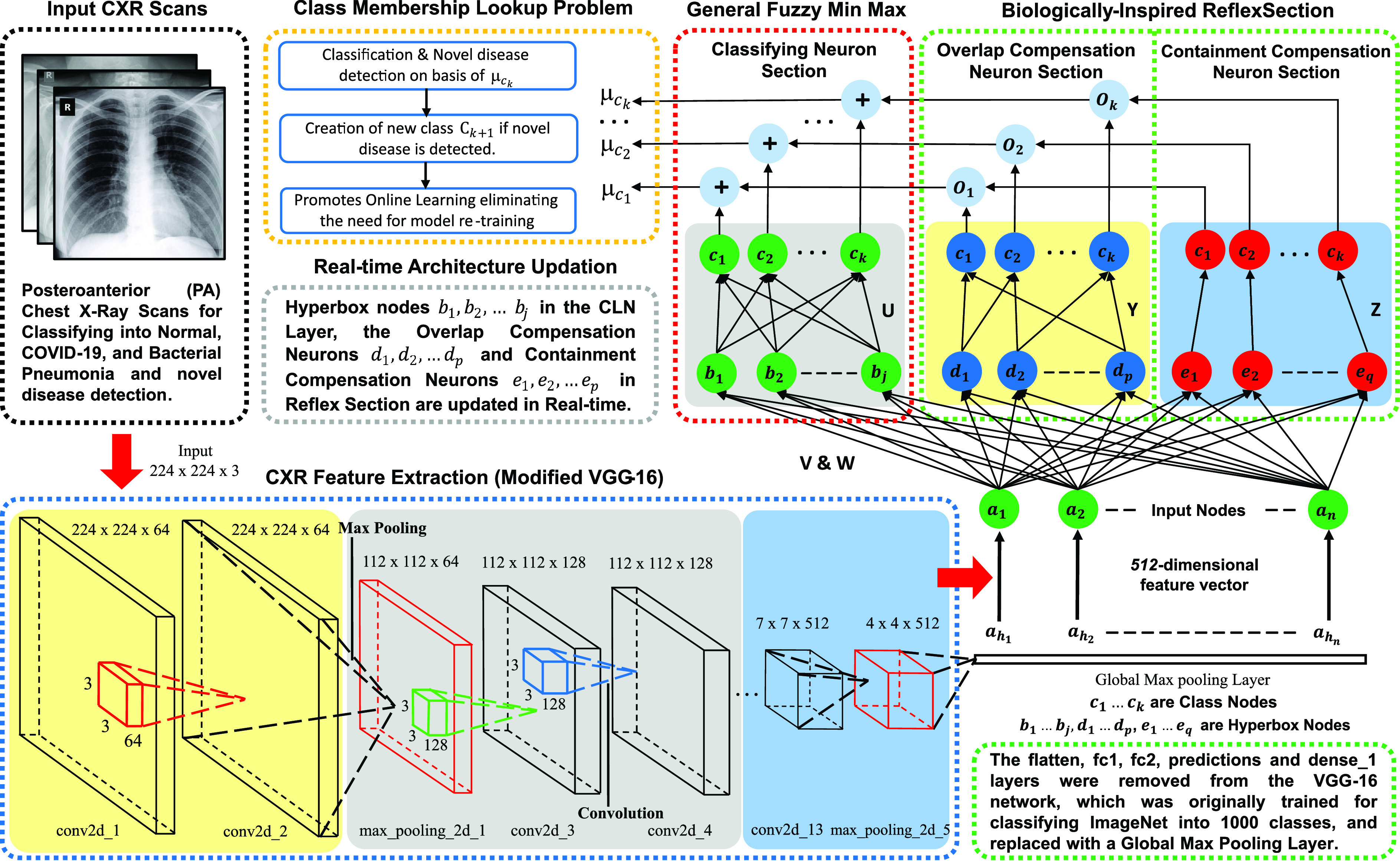
Architecture of the proposed Biologically-Inspired Conv-Fuzzy network. Here, Deep-Precognitive diagnosis is formulated as a class membership lookup problem.

The proposed model architecture (refer Figure 3) combines CNN-based CXR feature extraction with a fuzzy classification network for real-time image classification. From each sample, the image feature vector $a_{h} \in I^{n}$ containing prominent disease characteristics like ground-glass opacities, crazy paving pattern, etc., are extracted.

Deep transfer learning is used on modified VGG-16 [52] CNN architecture. A Global Max Pooling (GMP) layer is introduced after the fifth ‘MaxPooling2D’ layer of the original network, as shown in Table 2. Subsequent layers, i.e., the flatten, fc1, fc2, predictions, and dense_1 layers, are removed from the original network, which were primarily trained for classifying ImageNet [53] into 1000 different classes. Recent studies [54] have confirmed the effectiveness of transfer learning, which surprisingly offers higher accuracy in medical imaging tasks. Unlike standard dense (layer) in conventional CNNs, using fuzzy classification enables online learning and allows single-pass training compared to CNNs, which require multiple epochs to obtain high accuracy. Figure 5 shows the layer-specific feature representation maps generated by the feature extraction network.

**TABLE 2:** Architecture of the Modified VGG-16 CNN for Extracting 512-Dimensional CXR Feature Vector. Here, $S$ Represents Stage, $L$ is the Number of Stacked Layers in the Operator, $W/H/C$ Represents the Weight/ Height/ Channel and GMP is the Global Max Pooling Layer

S	Operator	Input $\rightarrow $ Output $(W \times H \times C)$	L
1	Conv3-64	$224\times 224\times 3\,\,\rightarrow \,\,224\times 224\times64$	2
2	MaxPooling2D	$224\times 224\times 64\,\,\rightarrow \,\,112\times 112\times64$	1
3	Conv3-128	$112\times 112\times 64\,\,\rightarrow \,\,112\times 112\times128$	2
4	MaxPooling2D	$112\times 112\times 128\,\,\rightarrow \,\,56\times 56\times128$	1
5	Conv3-256	$56\times 56\times 128\,\,\rightarrow \,\,56\times 56\times256$	3
6	MaxPooling2D	$56\times 56\times 256\,\,\rightarrow \,\,28\times 28\times256$	1
7	Conv3-512	$28\times 28\times 256\,\,\rightarrow \,\,28\times 28\times512$	3
8	MaxPooling2D	$28\times 28\times 512\,\,\rightarrow \,\,14\times 14\times512$	1
9	Conv3-512	$14\times 14\times 512\,\,\rightarrow \,\,14\times 14\times512$	3
10	MaxPooling2D	$14\times 14\times 512\,\,\rightarrow \,\,7\times 7\times512$	1
	GMP	$7\times 7\times 512\,\,\rightarrow \,\,1\times 1\times512$	1

**FIGURE 4.**
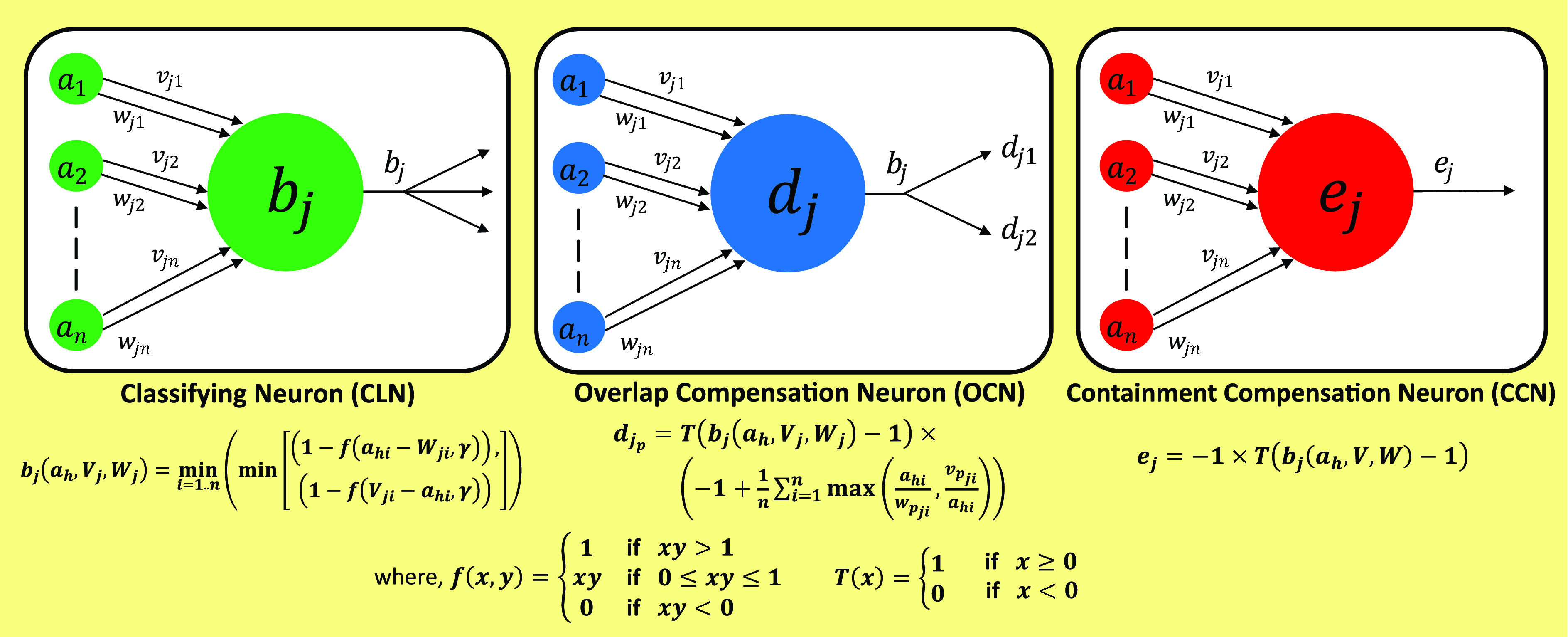
The three categories of Neuron (a) Classifying Neuron ( $CLN$) with its activation function $b_{j}$ (b) $OCN$ node and its activation function $d_{j_{p}}$ (c) $CCN$ node and its activation function $e_{j}$, used in the proposed model architecture; $f(x, y)$ and $T(x)$ represents the threshold functions.

**FIGURE 5.**
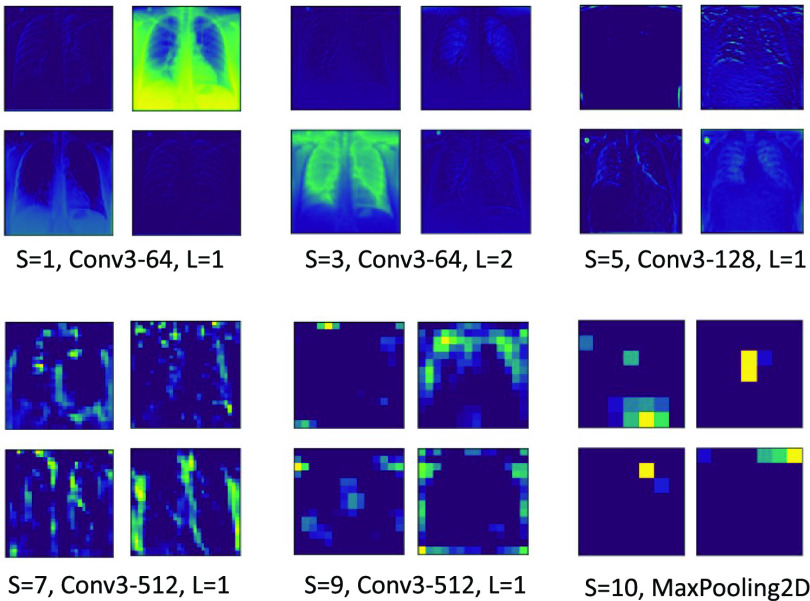
Layer-specific feature representation maps generated by the modified VGG-16 CXR feature extraction network.

### C. Fuzzy Classification

**FIGURE 6.**
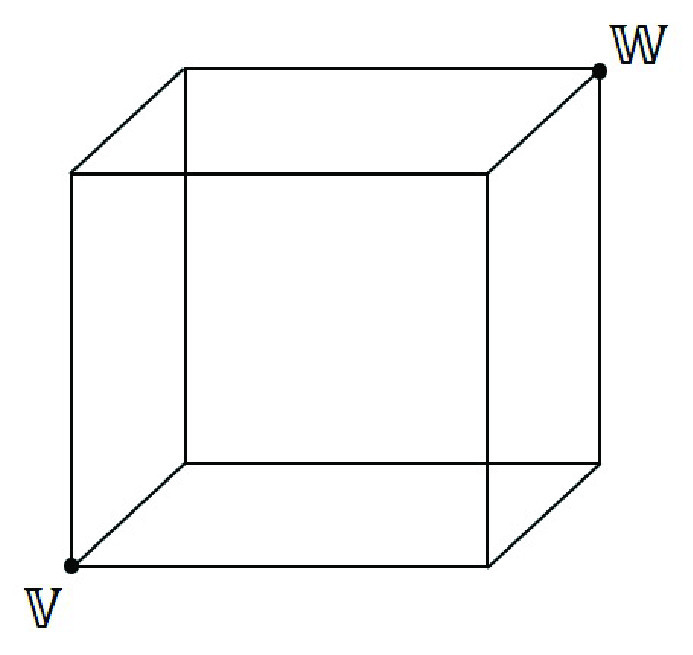
A Hyperbox $\mathcal {H}$ in 3-dimensional feature space i.e., $n=3$. Here, ‘ $V$’ represents min coordinate and ‘ $W$’ the max coordinate of $\mathcal {H}$.

The fuzzy classification network [46] learns by forming hyperbox fuzzy sets. A hyperbox $\mathcal {H}$ [55] is a simple geometrical-shape defined in the $n$-dimensional feature space. The size of $\mathcal {H}$ is controlled by the hyperbox expansion coefficient ( $\theta $), which lies between 0 and 1. Figure 6 shows a hyperbox $\mathcal {H}$ for $n=3$.

#### 1) Point Hyperbox ( $\mathcal{H}$) Creation

During training, the extracted feature vector $a_{h}$ for each training sample is passed to the input nodes $(a_{1},\ldots,a_{h})$ of the fuzzy classifier after normalization. The classifier creates hyperboxes with $\min $ co-ordinate $V_{j}=(v_{j1},v_{j2},\ldots,v_{jn})$ and $\max $ co-ordinate $W_{j}=(w_{j1},w_{j2},\ldots,w_{jn})$ in the 512-dimensional feature space. Assuming $\{a_{h}, C_{i}\}$ is the training sample, $\{b_{j},C_{j}\}$ is a hyperbox for class $C_{i}$; we initialize $\{b_{1}\}$ having $V_{1} = W_{1} = a_{h}$ and class label $C_{i}$, i.e., during training, for the first sample, a point hyperbox $\mathcal {H}$ is created.

#### 2) Class Membership Computation

For each sample, the extracted feature vector is passed to the Classifying Neurons ( $CLN$), for the classification of the learned data using $\min $- $\max $ hyperboxes [46]. A membership function [46] is defined with respect to the $\min $ and $\max $ co-ordinates of $\mathcal {H}$. In $CLN\text{s}$, neuron $b_{j}$ represents hyperbox fuzzy set $B_{j} (= A_{h},V_{j},W_{j},f(A_{h},V_{j},W_{j}) \,\,\forall \,\,(A_{h} \in I^{n}))$.

In classifying section nodes, to compute the class memberships, the activation function proposed by [56] is used to assign membership value equal to 1 when the test sample falls within $\mathcal {H}$. In other cases, when the test sample lies outside $\mathcal {H}$, the model calculates membership value based on its distance from extreme coordinates of $\mathcal {H}$. Figure 4(a) enlists the activation functions of the classifying neuron. Here, $f(x,y)$ represents a 2-parameter ramp threshold function and ‘ $\gamma $’ represents the fuzziness control parameter. It is to be noted that as the limit of the maximum allowed size of hyperbox ( $\theta $) increases, number of hyperboxes created during training reduces and vice-versa. This is confirmed experimentally in Section VI.

#### 3) Significance of Inter-Node Connections

In the middle layer of the classifier, the input nodes and the hyperbox nodes are connected together. These connections represents the $\min $- $\max $ coordinates $V$ and $W$ of the 512-dimensional hyperbox fuzzy set [48]. During training, the middle layer neurons are created dynamically. Connection between the hyperbox node $b_{j}$ to a class node $C_{j}$, is represented by matrix $U$, where, $u_{ij}=1$ if $b_{j} \in C_{j}$, else $u_{ij} = 0$.

#### 4) Training Learning Classifier

Whenever a training sample is encountered by the model that does not belong to the classes it has learned so far, a hyperbox node is created in the $CLN$ section. During training, the model tries to accommodate subsequent samples $\{a_{h},C_{i}\}$ in the previous hyperboxes belonging to the same class using the conditions discussed below, provided the hyperbox size does not exceeds a specified maximum limit (given by expansion coefficient $\theta $) [46]. If the expansion of any of the existing hyperboxes ( $\mathcal {H}$) which belongs to that class is not feasible, a new hyperbox is added to the model; i.e., for a new training sample $\{a_{h},C_{i}\}$, a hyperbox $\{b_{j}, C_{j}\}$ is found such that $C_{j}=C_{i}$ or $C_{j}=C_{0}$ which has the highest membership value and satisfies following conditions-

\begin{align*}&(1) ~\theta _{\max } \geq \frac {1}{n} \sum _{i=1}^{n} (\max (w_{ji}, a_{hi}) - \min (v_{ji}, a_{hi})) \tag{1}\\&(2) ~b_{j} {~\text {is not associated with any $OCN/ CCN$}} \tag{2}\\&(3) {~\text {if }} C_{i}=C_{0} {~\text {or }} C_{j}=C_{0} {~\text {then }} \mu _{j}>0, {~\text {where }} \mu _{j} \\&\qquad {~\text {is membership with hyperbox }} b_{j}, \tag{3}\end{align*}

Adjust min-max coordinates of $b_{j}$, as, $V_{ji}^{new} = \min (V_{ji}^{old}, a_{hi})$, $W_{ji}^{new} = \max (W_{ji}^{old}, a_{hi})$, where $i=1, 2, \ldots, n$ and if $C_{j} = C_{0}$ and $C_{i} \neq C_{0}$ then $C_{j} = C_{i}$.

If no suitable $b_{j}$ is present then a novel hyperbox $\mathcal {H}$ for class $C_{i}$ is created with $V_{j}=W_{j}=a_{h}$; i.e., a point hyperbox is created. Since the high dimensional feature space contains all the learned visual features of the image, a possible case of hyperbox overlap can occur. This can be explained as a common visual feature between two different diseases. Table 3 enlists both the common and distinguishing visual features on CXR scans for various diseases.

**TABLE 3:** Comparative Analysis of Visually Similar and Distinguishing Features of COVID-19, SARS-CoV-1 and MERS Respiratory Diseases on CXR Scans. Data Taken From [57]–[62]

Disease	Image Performance	Normal Radiography	Crazy Paving Pattern	Ground Glass Opacity	Consolidation	Septal Thickening	Air Bronchogram	Pleural Effusion	Pneumo-thorax
SARS-CoV	Unilateral, focal; unilateral, multifocal; bilateral; peripheral distribution	18.40%	46.27%	68.48%	65.65%	55.22%	37.04%	17.31%	9.62%
MERS-CoV	Bilateral, multifocal; isolated unilateral; peripheral distribution	20.00%	26.67%	86.36%	50.00%	40.91%	-	54.55%	Rare
COVID-19	Bilateral, multifocal peripheral distribution	19.90%	8.56%	68.92%	26.64%	34.54%	34.54%	3.57%	Rare

### D. Biologically-Inspired Reflex Section

The Reflex section contains the Overlap Compensation Neurons ( $OCN$) and Containment Compensation Neurons ( $CCN$) [48]. These neurons become active only when a case of hyperbox overlap and containment is encountered, respectively. The reflex mechanism is biologically inspired from that of the human brain, which unconsciously gains control of the human body in hazardous conditions.

#### 1) Reflex Section Architecture

$OCN$ represents $\mathcal {H}$ of size equal to the overlapped space between two $\mathcal {H}$ of different classes. The $OCN$ section is active only if the test data lies within the overlap space. It generates two compensation outputs, one each for the two overlapping classes. The $CCN$ section, which overcomes the hyperbox containment case, represents $\mathcal {H}$ of size equal to the overlapping space between the two classes. $CCN$ activates when a test sample falls inside the overlapped space. Figure 4 represents these nodes with their respective activation functions used in the model architecture.

#### 2) Inter-Node Connections and Training

The connection between the hyperbox nodes and class nodes in the reflex section is represented by matrix $Y$ and $Z$, respectively (refer Figure 3). Whenever a situation of overlap/ partial or full containment of $\mathcal {H}$ is encountered, the hyperbox node is created dynamically in the reflex section’s middle layer. Overlap or containment between a labeled hyperbox $(B_{j} \in C_{i}, \forall ~i > 0)$ and unlabeled hyperbox $(B_{k} \in C_{i}, \forall \,\,i = 0)$ is allowed and does not create any $OCN$ or $CCN$ nodes. This is used to label the unlabeled hyperboxes. The number of output layer nodes, present in the $CLN$ section, represents the total number of classes learned by the model.

#### 3) Final Membership Computation

The final membership value [48] for the $i^{th}$ class node is computated as $\mu _{i} \leftarrow C_{i} + O_{i}$, where $C_{i}$ is the membership of the $i^{th}$ class in classifying layer ( $CLN$) section; i.e., $C_{i} = \max \limits _{m = 1 \ldots j}(b_{m} u_{mi})$ and $O_{i}$ is the compensation $O_{i} = \min (\min \limits _{j = 1 \ldots p} (d_{j} y_{ji}), \min \limits _{j = 1 \ldots q} (e_{j} z_{ji}))$ for the $i^{th}$ class.

#### 4) Disease Visual Feature Overlap and Containment

**FIGURE 7.**
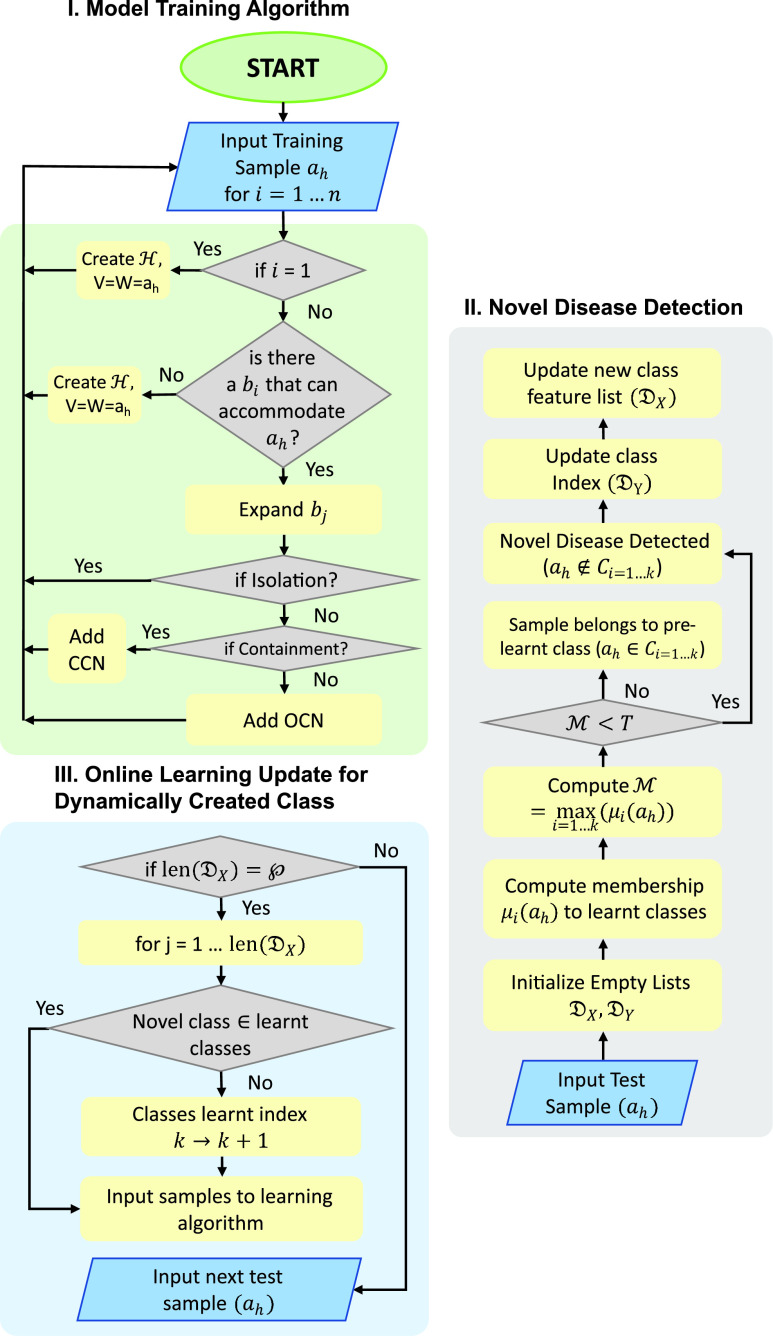
Algorithm Flowchart, I. Training Algorithm, II. Online learning for dynamically created class, III. Novel disease detection.

The introduction of this biologically-inspired section helps in obtaining more explainable class memberships. This is discussed in detail in the Ablation Study in Section IV. In the case of two diseases having similar visual features, a condition of hyperbox overlap may occur. Suppose a hyperbox $b_{j}$, which is expanded in any previous step, is compared with all other hyperboxes $b_{k}$. If $C_{j}$ and $C_{k}=C_{0}$, the overlap and contraction test are performed as explained in Test 2 [48]. They follow the principle of minimum disturbance by computing the dimension with minimum overlap ‘ $d$’ and contracting it. Otherwise, Test 1 is performed [48]. Figure 7 illustrates the algorithm as a flowchart.

#### 5) Hyperbox Isolation Condition

If $(V_{ki} < W_{ki} < V_{ji} < W_{ji})$ or $(V_{ji} < W_{ji} < V_{ki} < W_{ki})$ holds for any $i \in 1, \ldots, n$, then, $(b_{k}, b_{j})$ are isolated. If the condition does not hold, containment test is performed.

#### 6) Hyperbox Containment Condition

If $(V_{ki} < V_{ji} < W_{ji} < W_{ji})$ or $(V_{ji} < V_{ki} < W_{ki} < W_{ji})$ holds for any $i \in 1, \ldots, n$, then Hyperboxes are contained and a $CCN$ node is formed dynamically. If hyperboxes are not contained, an $OCN$ node is created.

#### 7) Hyperbox Overlap Test

Initial value of $\delta ^{old}$ is set as 1.

Case 1:
$v_{ji} < v_{ki} < w_{ji} < w_{ki}\,\,\delta ^{new}$ = $min(w_{ji} - v_{ki}, \delta ^{old})$

Case 2:
$v_{ki} < v_{ji} < w_{ki} < w_{ji}\,\,\delta ^{new}$ = $min(w_{ki} - v_{ji}, \delta ^{old})$

Case 3:
$v_{ji} < v_{ki} \leq w_{ki} < w_{ji}\,\,\delta ^{new}$ = $min(min(w_{ki} - v_{ji}, w_{ji} - v_{ki}), \delta ^{old})$

Case 4:
$v_{ki} < v_{ji} \leq w_{ji} < w_{ki}\,\,\delta ^{new}$ = $min(min(w_{ki} - v_{ji}, w_{ji} - v_{ki}), \delta ^{old})$

If overlaps exist and $(\delta _{new} - \delta _{old}) > 0$, then, $\Delta = i$ **else** $\Delta = -1$.

#### 8) Hyperbox Contraction Test

If overlap exists and is minimum along $\Delta $ dimension, the hyperboxes are contracted using the following given conditions:

Case 1:
$v_{j\Delta } < v_{k\Delta } < w_{j\Delta } < w_{k\Delta }\,\,v_{k \Delta }^{new}$ = $w_{j \Delta }^{new} = \frac {w_{j\Delta }^{old} + v_{k\Delta }^{old}}{2}$

Case 2:
$v_{k\Delta } < v_{j\Delta } < w_{k\Delta } < w_{j\Delta }\,\,v_{k \Delta }^{new}$ = $w_{j \Delta }^{new} = \frac {w_{k\Delta }^{old} + v_{j\Delta }^{old}}{2}$

Case 3:
$v_{k\Delta } < v_{j\Delta } \leq w_{j\Delta } < w_{k\Delta }$ and $w_{k\Delta } - v_{j\Delta } < w_{j\Delta } - v_{k\Delta }$ then $v_{j\Delta }^{new} = w_{k\Delta }^{old}$ else $w_{j\Delta }^{new} = v_{k\Delta }^{old}$

Case 4:
$v_{j\Delta } < v_{k\Delta } \leq w_{k\Delta } < w_{j\Delta }$ and $w_{k\Delta } - v_{j\Delta } < w_{j\Delta } - v_{k\Delta }$ then $w_{j\Delta }^{new} = v_{k\Delta }^{old}$ else $v_{j\Delta }^{new} = w_{k\Delta }^{old}$

### E. Novel Disease Detection System

This section describes how the model detects and classifies novel diseases, without explicit training.

#### 1) De Novo Disease Detection

For a particular disease, hyperboxes occupy a large extent of space in an $n$-dimensional feature space. For many diseases, since the type and extent of lesions tend to be either slightly or considerably similar, a portion of visual features is mapped to an $n$-dimensional feature space, which is common for more than one disease category. Most techniques tend to distinguish different disease classes on the basis of their differentiating visual features [51]. In the case of fuzzy classifiers, both common and differentiating features of the disease are mapped in the $n$-dimensional feature space using hyperboxes. Even though transfer learning is used to obtain feature vectors, since these are of high dimension, they encode a large amount of visual information of the disease [54]. Exploiting this property, we classify those diseases as novel which tend to occupy an overlapping space less than a set threshold $\mathcal {T}$, where $\mathcal {T} \in (0,1)$, in this $n$-dimensional feature space.

#### 2) Negation Operation on Intuitionistic Membership Grades

The regular membership function $\mu (x)$ denotes the value of an input sample belonging to a particular class of disease that CAD model has previously learned. The fundamental property, $\mu (x) + v(x) = 1$ and $\mu (x), v(x) \in [{0,1}]$, where $v(x)$ denotes the value by which the entity does not belong to class $C_{i}$. Using the negation operation, for intuitionistic membership grades, we tend to compute the input samples where $v(x)$ is above-set threshold $\mathcal {T}$, i.e, samples for which $\mathcal {M} < \mathcal {T}$, where $\mathcal {M}= \max \limits _{i = 1 \ldots k}(\mu _{i}(a_{h}))$. Widely used Pythagorean membership grade [63] defined by ${({(\mu (x))}^{2} + {(v(x))}^{2})}^{1/2} \leq 1$ is not employed since it allows for representation on a larger body of non-standard membership grades.

#### 3) Online Architecture Update for $C_{k+1}$ Class

Upon identifying a novel disease, the model adds a new class $C_{k+1}$ to the classification network, which is similar to class addition during model training. Further, when samples of this novel disease are detected in the future, the model constructs hyperboxes using the same principle discussed. This enables online architecture update and increases the model’s ability to classify the samples of the novel disease.

#### 4) Midway Labeled Data $(a_{h}, C_{i})$ Introduction

Furthermore, since the architecture is semi-supervised, labeled training samples of the novel disease can also be introduced at any point to improve the accuracy for this class without the need for complete re-training. Algorithm 1 is used for the detection of novel disease samples, online learning, and midway introduction of a labeled dataset. In detecting novel diseases, one of the challenges faced is determining accurate model parameters best suited for classification. Increasing the fuzziness control parameter ( $\gamma $) leads to more fuzzy classification while decreasing it leads to a crisp classification. Algorithm 1 discusses the implementation of the methodology in detail.

Algorithm 1 **Detecting and Classifying Novel Disease Occurrences**
Input and initialization: Model Pre-trained on labeled Train Set
$a_{h} \leftarrow $ Input test sample
$\theta $, $\gamma $, $\mathcal {T} \leftarrow $ Model Parameters
$k \leftarrow $ Current classes learnt
$\mathcal {D_{X}, D_{Y}} \leftarrow $ empty lists for feature vector, class index
$\mathcal {P} \leftarrow $User-input value for Online Learning
**Computing maximum class membership ( $\mathcal {M}$):**
$\mathcal {M}= \max \limits _{i = 1 \ldots k}(\mu _{i}(a_{h}))$, $\mathcal {M} \in (0,1)$
**Novel disease detection:**
**if** ( $\mathcal {M} < $ Threshold $\mathcal {T}$) **then**
$a_{h} \notin C_{i=1\ldots k}$, i.e., Novel Disease Detected
$\mathcal {D_{X}}$.append( $a_{h}$), $\mathcal {D_{Y}}$.append( $k+1$)
**else**
$a_{h} \in C_{i=1\ldots k}$, for which $\mu _{i}(a_{h})$ is $\max $
**Online Learning update for dynamically created class:**
**if** (elements in $\mathcal {D_{X}}$ equals $\mathcal {P}$) **then**
**for** ( $j \leftarrow 1$ to len( $\mathcal {D_{X}}$)) **do**
**if** ( $\mathcal {D_{Y}}$[ $j$] $\notin $ classes learned) **then**
Add $CCN$ Hyperbox $\mathcal {H}$
Classes learnt $\leftarrow (k+1)$, i.e., $C_{1}, C_{2}, \ldots, C_{k} \rightarrow C_{1}, C_{2}, \ldots, C_{k}, C_{k+1}$
**else**
Calculate expanded index
Follow standard algorithm
$\mathcal {D_{X}}$, $\mathcal {D_{Y}}$ = [], []
**Go to next test sample.**
**Midway introduction of labeled dataset:**
Train ( $a_{h}, C_{i}$), $i=1\ldots (k+1)$

## IV. Experiments and Results

A set of experiments are performed to demonstrate the effectiveness of the proposed approach. In this section, firstly, we discuss the experiments and the results of the model’s ability to detect novel diseases on three unseen novel disease datasets: SARS-CoV-1, MERS-CoV, and COVID-19 to demonstrate the strong generalizability of the approach. In additional experiments, the model’s classification ability is evaluated on two tasks: binary classification and multi-class classification of CXR diseases. We also discuss the hyperparameters chosen during these experiments. Lastly, we provide an ablation study to evaluate the contribution of key components of the proposed model.

### A. Experimental Settings

This section describes in detail the experimental setting.

#### 1) Dataset

To assess the proposed method, dataset from two popular open-source repositories- COVID-Chestxray set [64], and kaggle-chest-xray-dataset [65], extensively used in the research literature for training and testing of CAD models for COVID-19 CXR classification were employed. Table 4 contains the statistical details of posterior-anterior (PA) CXR scans used in the experiments. Initially, all inputs are pre-processed, which includes resizing $(224 \times 224 \times 3)$ and format conversion. Pre-processed high-quality images are selected and divided into two subsets: the training set (80%) and the test set (20%). The images are evenly distributed in different classes for classification experiments.

**TABLE 4:** Details of the CXR Radiograph Dataset Used to Assess the Proposed Method. The Proposed Model is Trained on Limited Dataset

	Disease Category	Ref	Total CXR Scans	Training Set (80%)	Test Set (20%)
1	COVID-19	[64]	196	157	39
2	Bacterial Pneumonia	[65]	196	157	39
3	SARS-CoV-1	[64]	08	-	08
4	MERS-CoV	[64]	08	-	08
5	Normal	[65]	196	157	39

#### 2) Implementation

The work is implemented using Keras [66] with Tensorflow [67] as backend. Nvidia K80 GPU with 12GB RAM workbench was used for conducting the experiments. ‘zscore’ was used as the normalization method which is calculated as $z = (x - u) / s$. ‘yeo-johnson’ transformation was applied while training the ML classifiers for comparison.

#### 3) Compared Methods

Various SOTA ML classifiers are implemented on the same dataset to compare the classification results. 15-fold cross-validation was used for implementing the classifiers to distinguish between COVID-19 and non-COVID-19 CXR scans compared to the proposed model on the same dataset. Similarly, the models were implemented for performance comparison on the multi-class classification task. ‘Accuracy’ was used as the metric for optimizing the hyperparameters used for training.

#### 4) Evaluation Metrics

Confusion matrix-based metrics is used assess the classification performance of the proposed model. This includes accuracy, precision, recall, and F1-score as described below.

1) **Accuracy:** It estimates the ratio of correctly classified diseases to the entire test dataset. If accuracy is higher, a model has better performance. It lies between [0, 1] and is generally reported as percentage (%).
\begin{equation*} Accuracy = \frac {N_{TN} + N_{TP}}{N_{TN} + N_{FN} + N_{TP} + N_{FP}}\tag{4}\end{equation*}
2) **Precision:** It estimates the ratio of a particular disease that has been correctly classified over CXR scan to the total number of that particular disease identified by the model. Like accuracy, precision also lies between [0, 1] and is generally reported as a percentage (%).
\begin{equation*} Precision = \frac {N_{TP}}{N_{TP} + N_{FP}}\tag{5}\end{equation*}
3) **Recall:** The recall is the measure of a model correctly identifying true positives. Thus, for all the patients who actually have a particular disease over CXR scan, recall tells how many were correctly identified having that particular disease.
\begin{equation*} Recall = \frac {N_{TP}}{N_{TP} + N_{FN}}\tag{6}\end{equation*}
4) **F1-Score:** It is defined as the harmonic mean of Precision and Recall. If the F1-Score is higher, a model is better. F1-Score $\in [{0, 1}]$ and is generally reported as percentage (%).
\begin{equation*} F1-Score = 2\times \left({\frac {Precision \times Recall}{Precision+Recall}}\right)\tag{7}\end{equation*}

where, $N_{TP}$, $N_{TN}$, $N_{FP}$, $N_{FN}$ are the number of true positives, true negatives, false positives and false negatives respectively. For multi-class classification task, the discussed metrics is used as class-wise and macro-average.

### B. Exp 1: Evaluation of Novel Disease Detection Ability

In this experiment, the model is trained to classify normal X-ray scans from bacterial pneumonia X-rays. To evaluate the ability of the model to detect novel diseases, CXR samples of diseases, like SARS-CoV-1, MERS-CoV, and SARS-CoV-2 (COVID-19), not seen by the model before, are inputted along with the original test set images.

**FIGURE 8.**
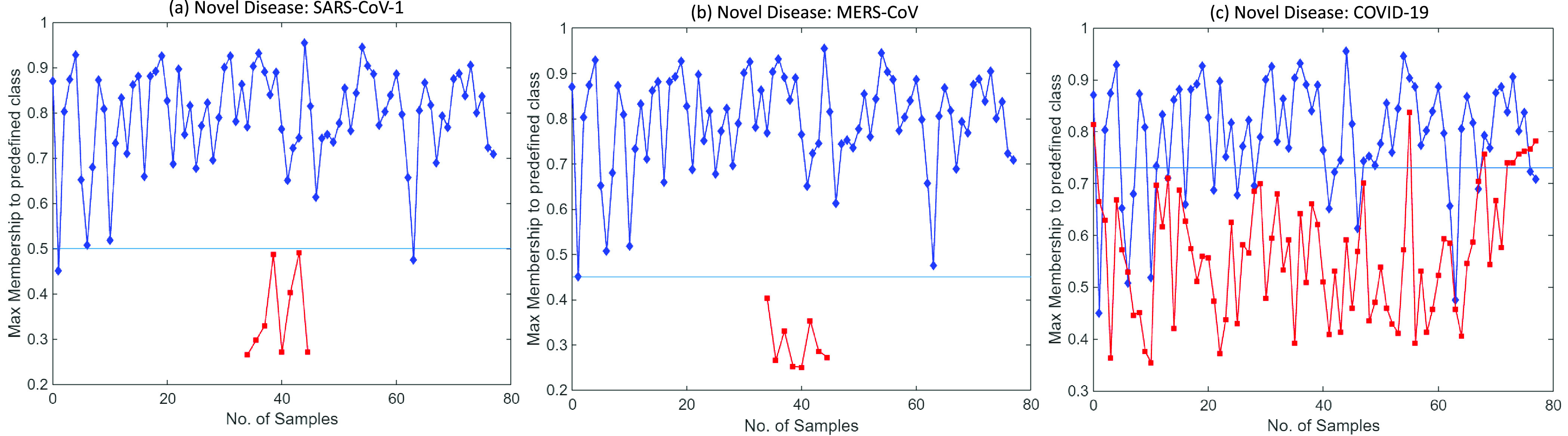
Graph between max membership to predefined class $\mathcal {M}$ and number of input test samples for (a) SARS-CoV-1, (b) MERS-CoV and (c) SARS-CoV-2 datasets. Unseen novel disease samples detected by the proposed model and classified separately in a newly created class. Here, max memberships to predefined class, i.e., $\mathcal {M}$ for the novel disease samples are in red and to classes learned previously are in blue.

Severe acute respiratory syndrome (SARS-CoV-1) is a viral respiratory disease reported around the end of February 2003. SARS-CoV-1 samples are used as novel disease sample inputs to the proposed model along with the test set. Figure 8(a) shows the graph obtained (at $\theta =0.75$ and $\gamma =1$) between the max-memberships value to the pre-defined class; i.e., $\mathcal {M}$ for different input test samples $\{a_{h}\}$. It can be inferred from the graph that samples belonging to the classes that the model was pre-trained for, i.e., normal and bacterial pneumonia, have a higher range of membership values over predefined classes. However, the input test samples of novel disease are found to have max class memberships below a certain set threshold. This is due to different visual features than those of the previously learned diseases. CXR scans of SARS-CoV-1 patients show bilateral airspace consolidation (observed in 66.7%–70.6% patients), demonstrated by multi-focal opacity [68]. Focal opacity is also detected predominantly in the middle, lower and peripheral zones of the lungs. This is significantly different from COVID-19 and MERS-CoV, where consolidation is 26.64% and 50% respectively, as compared to 65.65% in SARS-CoV-1 (refer Table 3). The SARS-CoV-1 input samples are classified into a new dynamically created class during testing with $\theta =0.75$, $\gamma =1$ and threshold $\mathcal {T}=0.50$. Moreover, each time this new disease is further encountered in the future, the model classifies it to the newly created class.

**FIGURE 9.**
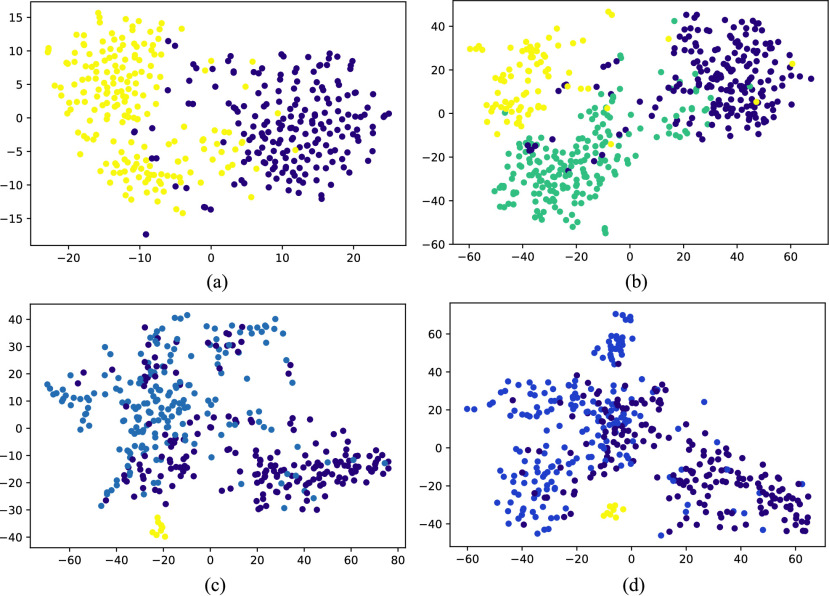
Visual illustration of $t-SNE$ feature visualization. (a) Normal vs Bacterial Pneumonia classification. Here yellow represents normal samples whereas Violet shows the Bacterial Pneumonia samples. (b) COVID-19 (in green) is input as novel disease, previously unseen by the model. (c) MERS (in yellow) and (d) SARS (in yellow) is input as novel disease, previously unseen by the model. In both (c) and (d) green represents the normal CXR scans whereas Violet shows the Bacterial Pneumonia samples.

Similarly, to demonstrate the model’s generalization ability, MERS-CoV and COVID-19 CXR scans are used with the proposed model as novel disease samples. Note that the model has not seen these images before and has never been trained on them. The model detects them as novel diseases, classifying them to a newly created class. Figures 8(b) and 8(c) show the graph obtained between max-memberships value $\mathcal {M}$ to pre-defined class for MERS-CoV and COVID-19. Further, Figure 9 shows the results obtained from t-Distributed Stochastic Neighbor Embedding (t-SNE) feature visualization for the Normal vs Bacterial Pneumonia classification and the *de novo* disease detection experiments with CXR of COVID-19, MERS-CoV and SARS-CoV-1 input as novel disease. The performance of the model, along with the hyperparameters used, are discussed in Table 5.

**TABLE 5:** Performance of the Proposed Model on COVID-19, SARS-CoV-1 and MERS-CoV Datasets for Novel Disease Detection Experiment Along With Hyperparameters $\theta, \gamma, \mathcal{T}$ Used. The Model was Pre-Trained to Classify Normal and Bacterial Pneumonia Classes

Novel disease (unseen by the model)	$\mathcal {\theta }$	$\mathcal {\gamma }$	${\mathcal {T}}$	Novel Disease Detection Acc. ( $\uparrow $)	Bacterial Pneu. vs Normal CXR classification Acc. ( $\uparrow $)
COVID-19	0.75	1	0.70	82.50%	81.01%
SARS-CoV-1	0.75	1	0.50	100.00%	96.20%
MERS-CoV	0.75	1	0.45	100.00%	98.73%

### C. Exp 2: Evaluation of Classification Ability

In this experiment, the proposed model is tested to assess its performance on binary and multi-class classification tasks to demonstrate that the model is not only capable of detecting *de novo* diseases but also classifying test samples belonging to other pre-trained classes $C_{i=1\ldots n}$. Binary classification is performed on non-COVID-19 and COVID-19 CXR scans, whereas multi-class classification is performed between normal, bacterial pneumonia and COVID-19 CXR samples.

**FIGURE 10.**
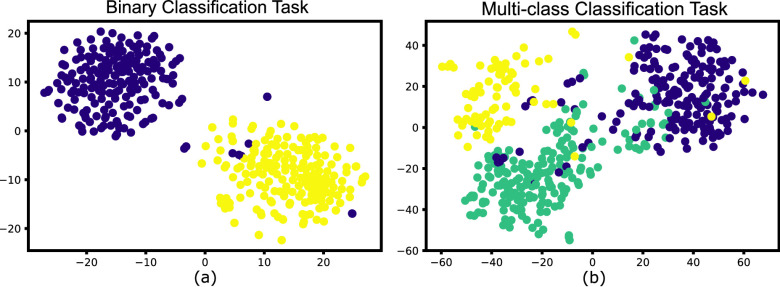
Visual illustration of $t-SNE$ feature visualization for (a) Normal vs COVID-19 binary classification task. Here yellow represents COVID-19 samples, whereas normal samples are shown in Violet (b) Normal vs COVID-19 vs Bacterial Pneumonia multi-class classification task. Here green shows the Bacterial Pneumonia class.

**FIGURE 11.**
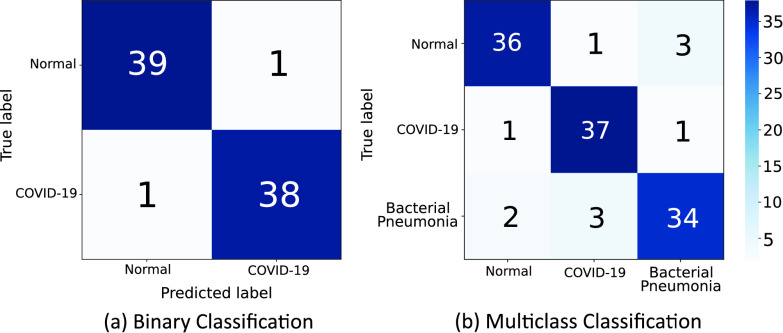
The obtained Confusion matrix for the (a) Binary classification and (b) multi-class classification task.

The obtained results are compared with the various ML classifiers implemented on the same dataset. Table 6 shows the 15-fold cross-validation performance of SOTA ML classifiers to distinguish between COVID-19 and non-COVID-19 CXR scans compared to the proposed model. Table 7 compares the performance of the proposed model with various ML classifiers on the multi-class classification task. The results demonstrate that the performance of the proposed model is at par with other SOTA models in both classification tasks. Figure 11 illustrates the obtained confusion matrix for both the classification tasks. Further, Figure 10 shows the results obtained from the t-SNE feature visualization for both the classification tasks.

**TABLE 6:** Comparison of Binary Classification Results on CXR Scans With Various ML Classifiers. Here, $\theta= 0.85$, $\gamma= 2$ for Proposed Method. Feature Vectors are Obtained From CXR Feature Extraction Network

Method	Accuracy ( $\uparrow $)	Recall ( $\uparrow $)	Precision ( $\uparrow $)	F1 Score ( $\uparrow $)	TT (sec) ( $\downarrow $)
Quadratic Discriminant Analysis (QDA)	65.38	59.26	67.15	62.10	0.0507
Linear Discriminant Analysis (LDA)	78.85	80.74	79.26	77.82	0.0747
Gradient Boosting Classifier (GBC)	93.41	93.33	93.76	93.31	1.7200
Decision Tree (DT)	95.98	97.04	95.31	96.01	0.0467
Random Forest (RF)	96.32	97.04	95.78	96.33	0.5780
Extreme Gradient Boosting (XG-Boost)	96.35	96.30	96.52	96.24	1.7660
Support Vector Machine (SVM)	97.06	97.78	96.71	97.11	0.0400
Naive Bayes (NB)	97.06	100.0	94.91	97.26	0.0280
Proposed Method	97.47	97.46	97.46	97.46	0.7878

**TABLE 7:** Comparison of Multi-Class Classification Results on CXR Scans With Various ML Classifiers. Here, $\theta= 0.60$, $\gamma= 1$ for Proposed Method. Feature Vectors are Obtained From CXR Feature Extraction Network

Method	Accuracy ( $\uparrow $)	Recall ( $\uparrow $)	Precision ( $\uparrow $)	F1 Score ( $\uparrow $)	TT (sec) ( $\downarrow $)
Quadratic Discriminant Analysis (QDA)	42.78	57.14	43.94	41.96	0.0480
Linear Discriminant Analysis (LDA)	63.75	64.07	65.11	63.07	0.0907
Decision Tree (DT)	76.13	76.10	77.61	76.00	0.0793
Naive Bayes (NB)	89.48	89.59	90.06	89.48	0.0253
Gradient Boosting Classifier (GBC)	90.51	90.64	91.21	90.47	8.4060
Proposed Method	90.68	90.70	90.67	90.63	5.7184
Extreme Gradient Boosting (XG-Boost)	91.72	91.76	92.30	91.70	10.817
Random Forest (RF)	91.95	91.93	92.55	91.95	0.6253
Support Vector Machine (SVM)	93.20	93.26	93.87	93.16	0.0700

### D. Ablation Studies

The proposed model contains four key components: The modified VGG-16 based CXR feature extractor, fuzzy classifier, biologically-inspired reflex section for class membership generation, and the novel disease detection framework. Here, we provide an ablation study to explore the contribution of the key components of the proposed model.

#### 1) Global Max-Pooling vs. Global Average Pooling

Although pneumonia and most other diseases can be differentiated, X-rays are still considered as the “most challenging plain film to interpret correctly” [33]. Therefore, extracting discriminating features on CXR scans is a challenging task. Instead of using the standard global average pooling (GAP) layer in the feature extraction network, the global max-pooling layer (GMP) is preferred. During the ablation study, the GMP layer was replaced with the GAP layer to study its contribution. Unlike conventional models, in which GAP outperforms GMP, in the present model, GMP is found to generate more representable features from the input image and shows significant performance over GAP. This mainly occurs when visual features of two image classes are very near to each other with very minute differences. Therefore, in such cases averaging the features, i.e., using GAP fails.

#### 2) Varying CNN Models and Curse of Dimensionality

The model is found to perform optimally when CXR feature vector dimensionality $n=512$. As the dimensionality is increased above 512, the model accuracy decreases, and the sample testing time is also found to increase significantly. This is due to multi-fold increase in the volume of the high dimensional space, such that the available data becomes sparse. Further, modified VGG-16 [52] CNN pre-trained on ImageNet [53] performs optimally over other feature extraction networks like ResNet [69], MobileNet [70], etc.

#### 3) Explainability of Class Memberships

Introduction of biologically-inspired section helps in obtaining more explainable class memberships. Unlike fuzzy min-max neural networks [46], which contracts an hyperbox in case of overlaps, the reflex mechanism produces compensation outputs using $OCN$ and $CCN$ neurons. This brings the model near reality since most diseases have some visually similar features on CXR scans lost in FMNN due to hyperbox contraction.

## V. Discussion

### A. Parametric Study & Hyperparameter Tuning

An in-depth parametric study was performed to evaluate the effects of various model parameters and propose a strategy for hyperparameter tuning. Figure 14 shows plots obtained from the parametric study; i.e., the effect of various parameters including the hyperbox expansion coefficient ( $\theta $) and fuzziness control parameter ( $\gamma $) on the model accuracy, number of hyperboxes ( $\mathcal {H}$) created during model training, total model training time ( $sec$) and the sample testing time ( $sec$) for both the classification tasks performed to evaluate the model’s classification ability. The obtained results are:

1) At higher values of hyperbox expansion coefficient ( $\theta \geq 0.6$), the model shows better performance on image classification tasks (refer Figure 14(a-b)). In classification tasks where the feature vectors are mapped to low-dimensional space, i.e., $n< 512$, low values of expansion coefficient ( $\theta $) produces optimal results.
2) Further, it can be inferred from Figure 14(a-b) that decreasing the fuzziness control parameter ( $\gamma $) generally shows better model performance.
3) As hyperbox expansion coefficient ( $\theta $) increases, the number of hyperboxes created during training shows an ‘exponential’ increase rather than ‘linear’ (refer Figure 14(g)). On the other hand, the model training time first shows a sharp rise until $\theta \approx 0.2$, after which its value decreases ‘exponentially’ in both the binary and multi-class classification tasks (refer Figure 14(h)).

**FIGURE 12.**
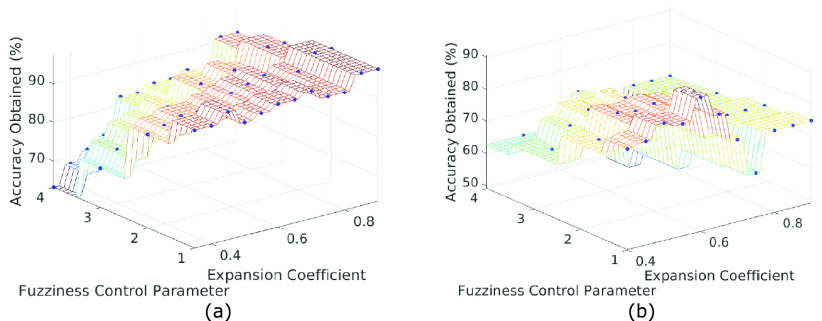
Graph between the obtained accuracy, fuzziness control parameter ( $\gamma $) and expansion coefficient ( $\theta $) to find the best fit model for (a) Binary Classification (b) Multi-class Classification tasks.

**FIGURE 13.**
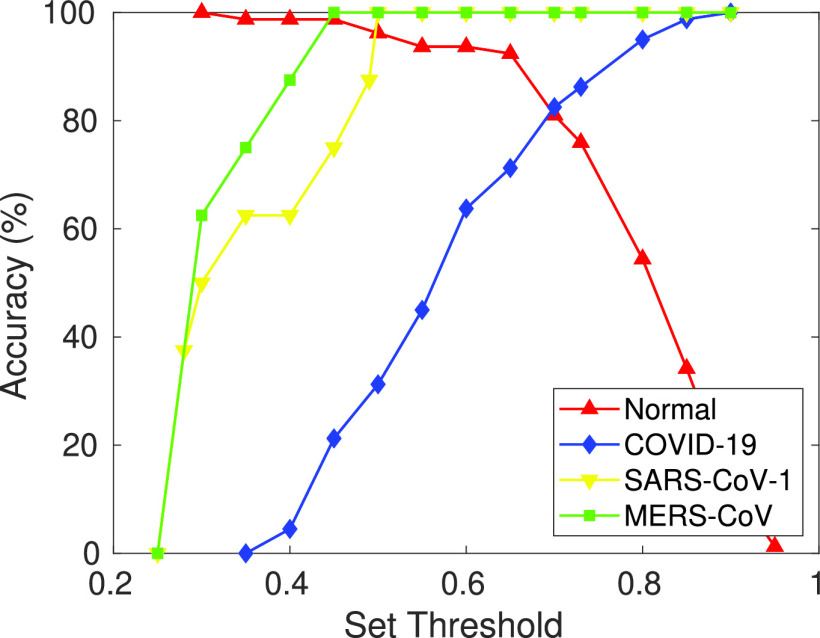
Graph obtained between accuracy and set threshold ( $\mathcal {T}$). The intersection point represents the best fit point.

**FIGURE 14.**
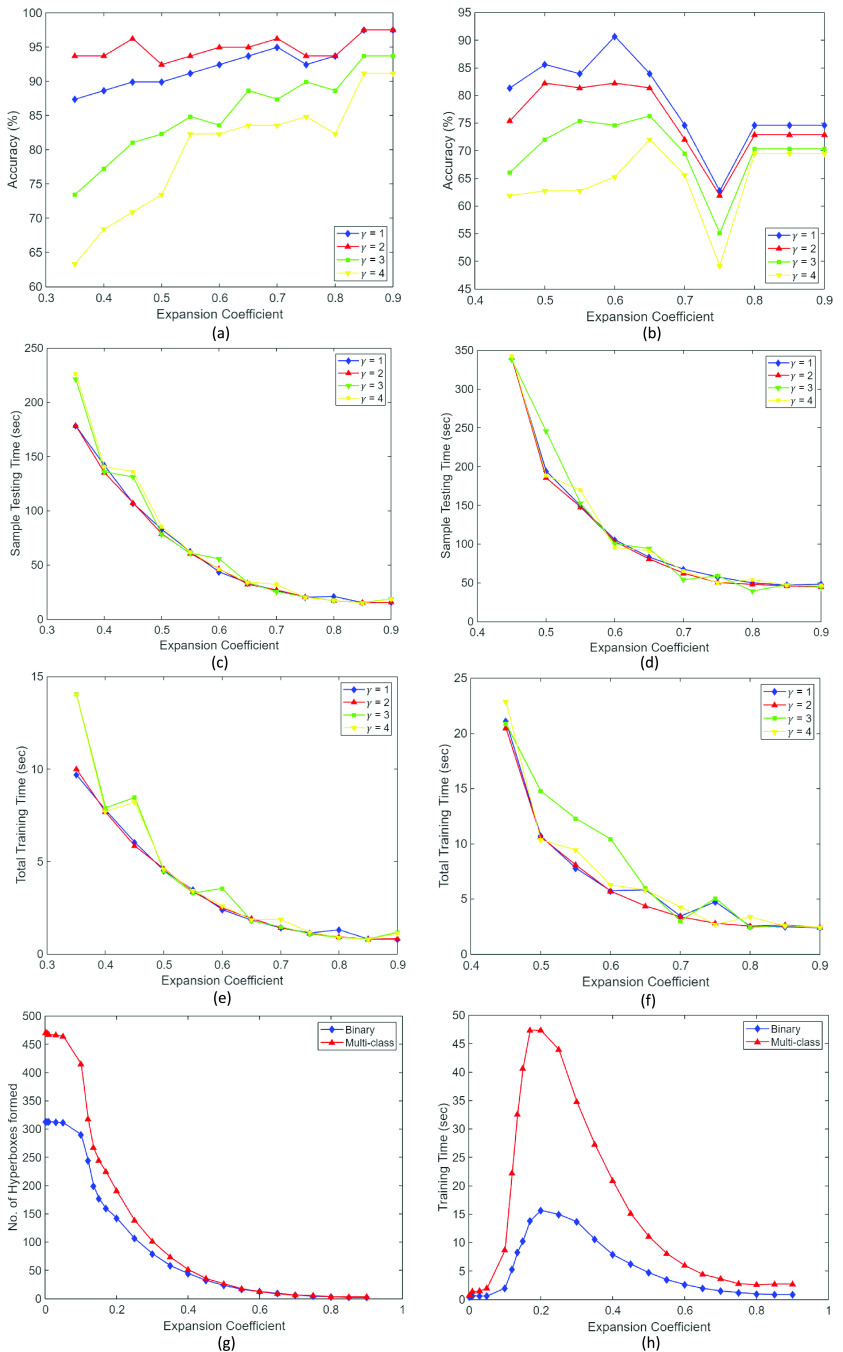
Plots obtained from parametric study. Graph between (a) Accuracy (%) and expansion coefficient ( $\theta $) for $\gamma = 1, 2, 3, 4$ for binary classification (b) Accuracy (%) and expansion coefficient ( $\theta $) for $\gamma = 1, 2, 3, 4$ for multi-class classification (c) Sample testing time (sec) and expansion coefficient ( $\theta $) for binary classification (d) Sample testing time (sec) and expansion coefficient ( $\theta $) for multi-class classification (e) Total training time (sec) and expansion coefficient ( $\theta $) for binary classification (f) Total training time (sec) and expansion coefficient ( $\theta $) for multi-class classification (g) Number of hyperboxes formed and expansion coefficient ( $\theta $) (h) Training time sec) and expansion coefficient ( $\theta $).

The results obtained from the study quantifies the model hyperparameters for image classification tasks and are helpful in hyperparameter tuning. Figure 12 shows a three-dimensional plot between the obtained classification accuracy, hyperbox expansion coefficient ( $\theta $), and the fuzziness control parameter ( $\gamma $) for both the classification tasks, carried out to find the best fit model.

The set threshold ( $\mathcal {T}$) plays a significant role in the detection of *de novo* diseases. Figure 13 compares the accuracy vs. set threshold in this regard. For class memberships, the threshold ( $\mathcal {T}$) is determined experimentally. For SARS-CoV-1, a threshold ( $\mathcal {T}$) is set at 0.5, MERS-CoV at 0.45, and 0.70 for COVID-19. The best fit values of the thresholds, obtained from the graph shown in Figure 13, are the point of intersection between the classification accuracy and class memberships for the novel disease. It can be inferred from the plot that when the threshold ( $\mathcal {T}$) for the novel disease is kept very low, novel disease samples are classified as one of the previously trained classes. However, when the threshold is kept very high, input samples belonging to the pre-trained classes are incorrectly classified as novel disease samples.

### B. Time Complexity Analysis

Along with the parametric study, a time complexity analysis is performed. In this study, the sample testing time (sec) is calculated by varying the hyperparameters $\theta $ and $\gamma $ for both the binary and multi-class classification tasks. The experiment is repeated to analyze the total model training time (refer Figure 14(c-f)). The obtained results quantified that though the total training time of the model is extremely less (i.e., $\approx ~5$ to 20 sec), the sample test time is relatively high and varies from $\approx ~50$ to 300 sec. The same is observed for both the binary and multi-class image classification tasks. In the case of low-dimensional data classification tasks, such a large difference is not observed.

### C. Robustness to Adversarial Attacks

Adversarial attacks [84] involves generating modified image by making subtle imperceptible changes in the original image. To boost robustness, existing defensive measures include: leveraging network distillation to extract information from the trained feature extractor [85], and using innovative training methods (such as IMA) that can expand the margins of training samples in the input space [86]. Since the feature extraction network was pre-trained on ImageNet [53], adversarial noise may not significantly affect the performance of the network. The model performance establishes strong generalizability of the approach through tests for a set of three novel diseases (taken from two different open-source datasets [64], [65]): COVID-19, SARS-CoV-1 and MERS-CoV. The main adversarial attack algorithms identified to target the proposed model include the L-BFGS algorithm and Fast gradient sign method (FGSM) [82]. Moreover, quantitatively analysing the vulnerability of the model to possible adversarial attacks w.r.t. existing defense method suitability, along with demonstrating robustness on various adversarial attacks can be an interesting extension for this research.

### D. State-of-the-Art Performance Comparison

**FIGURE 15.**
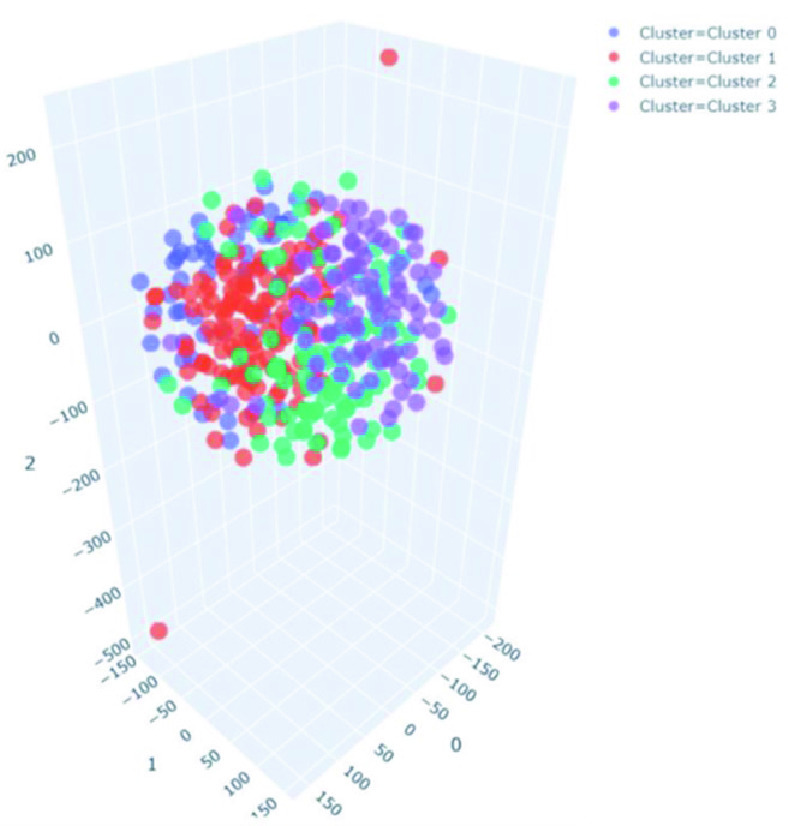
Results of the k-means clustering showing 04 identified clusters, depicting the failure of conventional clustering approaches.

To compare the performance of the proposed model with unsupervised clustering approaches, K-means clustering was performed on the dataset with normal, bacterial pneumonia, and COVID-19 X-ray feature vectors. The feature vectors are extracted using the same feature extraction network as used in the proposed model. K-means clustering was found to incorrectly detect 4 clusters as shown in t-SNE plot [83] in Figure 15. It illustrates the failure of conventional clustering approaches and how semi-supervised fuzzy classifiers are effective. For hyperbox expansion coefficient $\theta =0$, the fuzzy classifier is the k-nearest neighbor classifier.

A comparative analysis of the proposed model was performed with existing SOTA techniques developed employing CXR images. Table 8 summarizes the study’s findings. It shows both quantitatively and qualitatively the out-performance of the proposed model over other models in present literature. Moreover, the present work is the first to identify the challenging task of deep-precognitive diagnosis and propose a novel solution to it.

**TABLE 8:** Performance Comparison With SOTA Techniques in Literature Developed Using CXR Images. The Present Work is First to Identify the Challenging Task of Novel Disease Detection and Propose a Novel Solution to it

Study	Number of class/ Samples	Method	Accuracy (%)	Online Learning	Novel Disease Detection
Sahinbas & Catak [71]	2-class/ 140 (70 each of COVID-19 & Normal)	VGG16, VGG19, ResNet, DenseNet, InceptionV3	80.00	$\mathbf {N}$	$\mathbf {N}$
Panwar et al. [72]	2-class/ 570 (206 COVID-19 & 364 Normal)	CNN Transfer Learning	89.47	$\mathbf {N}$	$\mathbf {N}$
Hemdan et al. [73]	2-class/ 50 (25 each of COVID-19 & Normal)	COVIDX-Net	90.00	$\mathbf {N}$	$\mathbf {N}$
Medhi et al. [74]	2-class/ –	Deep CNN	93.00	$\mathbf {N}$	$\mathbf {N}$
Waheed et al. [75]	2-class/ 1124 (403 COVID-19, 721 Normal)	CovidGAN	95.00	$\mathbf {N}$	$\mathbf {N}$
Ahishali et al. [76]	2-class/ 13609 (1065 COVID-19, 1254 Normal)	CSEN	95.13	$\mathbf {N}$	$\mathbf {N}$
Vaid et al. [77]	2-class/ 545 (181 COVID-19, 364 Normal)	VGG16 Transfer Learning	96.30	$\mathbf {N}$	$\mathbf {N}$
Lv et al. [78]	2-class/ 270 (105 COVID-19 & 165 Normal)	Cascade Network	97.14	$\mathbf {N}$	$\mathbf {N}$
Proposed Study	2-class/392 (196 each of Normal & COVID-19)	Proposed Model	97.47	$\mathbf {Y}$	$\mathbf {Y}$
Civit-Masot et al. [79]	3-class/ 396 (132 each COVID-19, pneumonia, healthy)	VGG16 based DL model	86.00	$\mathbf {N}$	$\mathbf {N}$
Ozturk et al. [80]	3-class/ 1125 (125 COVID-19, 500 Normal, 500 Pneumonia)	DarkCovidNet	87.02	$\mathbf {N}$	$\mathbf {N}$
M. Qjidaa et al. [81]	3-class/ 300 (100 COVID-19, 100 Normal, 100 Pneumonia)	VGG-16 based model	87.50	$\mathbf {N}$	$\mathbf {N}$
Proposed Study	3-class/588 (196 each of Normal, Pneumonia & COVID-19)	Bio-Inspired Conv-Fuzzy Net	90.68	$\mathbf {Y}$	$\mathbf {Y}$
Proposed Study	392 (196 Normal & 196 Bacterial Pneumonia)	Proposed Model	–	$\mathbf {Y}$	COVID-19 82.5% SARS-CoV-1 100% MERS-CoV 100%

## VI. Limitations and Future Work

One of the limitations of the proposed model is that its sample testing time (i.e., $\approx 50$ to 300 sec) is comparatively higher than the total model training time (i.e., $\approx 5$ to 20 sec). Future work may look into ways to reduce the high sample testing time of the model. *Second*, an algorithm can be developed to avoid manual interventions for updating the hyperbox expansion coefficient ( $\theta $) and fuzziness coefficient ( $\gamma $). Moreover, we aim to increase the model sensitivity to novel diseases and improve classification accuracy by modifying the model architecture in future work. Future research directions also include expanding the proposed model over other diseases that are detectable through CXR features in high-dimensional vector spaces. Although in the future, there may be disease outbreaks that might impact some other human organ that can only be caught by scanning a particular organ or through blood chemistry, physiological analysis, CT-Scans, MRI, etc. Nevertheless, the proposed model can be further extended by changing or augmenting this kind of data. Future research in such areas will slowly help us move towards a universal DPD model that can take different kinds of data and predict new disease existence.

## VII. Conclusion

Currently, most works on DL-based CAD models are limited to increasing classification accuracy and sensitivity. In this paper, the concept of Deep-Precognitive diagnosis is proposed, which has immense potential for future research and can be helpful in the development of next-generation CAD models. The challenges posed by the formulated Deep-Precognitive diagnosis task are difficult to be addressed using supervised learning models, as they require labeled data for learning to classify new data into one of the trained classes only. Since it is not possible to get the novel disease dataset prior to its outbreak, most supervised learning models would fail to detect the new disease class.

The present work is the *first* to address these challenges by proposing a biologically-inspired convolutional fuzzy classification model, wherein we visualize the proposed task as a class membership lookup problem. Unlike conventional models, the proposed DPD model can detect occurrences of new diseases at their preliminary stage of spread. Such a model has the capability to control the spread of novel diseases with an early warning of a future pandemic. The proposed model creates a new class for them in real-time. Experimental results on three CXR disease data- SARS-CoV-1, MERS-CoV, and COVID-19 demonstrate the feasibility and remarkable performance in identifying a new disease class. Further, the model’s classification ability is demonstrated in the binary and multi-class classification tasks. An ablation study is also performed to quantify the contributions of critical components of the model. Thus, the proposed model can be used as a baseline for future works. Besides, two other vital issues that obstruct the development of future diagnosis models are also addressed in this work: firstly, the requirement of a large labeled training medical dataset and, secondly, the need for model re-training when novel disease needs to be added to the CAD model for classification. The model learns on limited datasets and reduces heavy dependence on labeled dataset availability. The results also establish that modified fuzzy classifiers achieve accuracy comparable with SOTA models.

Deep-Precognitive diagnosis has immense potential applications in developing future-CAD models that will be powerful enough to detect new disease occurrences and learn and improve their novel disease classification ability to expand on several such new diseases in real-time.

## Conflicts of Interest

The authors hereby declare that there is no conflict of interest.
